# Leptin receptor in the chicken ovary: potential involvement in ovarian dysfunction of ad libitum-fed broiler breeder hens

**DOI:** 10.1186/1477-7827-2-72

**Published:** 2004-10-08

**Authors:** Sandrine Cassy, Sonia Metayer, Sabine Crochet, Nicole Rideau, Anne Collin, Sophie Tesseraud

**Affiliations:** 1 Institut National de la Recherche Agronomique, Station de Recherches Avicoles, 37380 Nouzilly, France

## Abstract

In hens, the ovarian follicles committed to ovulation are arranged in an ordered follicular hierarchy. In standard broiler breeders hens genetically selected for high growth rate the reproductive function is clearly dysfunctional. Feed restriction is needed during reproductive development to limit the formation of excessive numbers of ovarian yellow follicles arranged in multiple hierarchies. To determine whether leptin is involved in the nutritional and reproductive interactions controlling follicular hierarchy in hens, blood leptin levels and ovarian expression of the leptin receptor mRNA were determined during follicle maturation in three chicken lines; a slow growing broiler "Label" genotype without reproductive dysfunction, a fast growing "Standard" genotype fed ad libitum or restricted and a fast growing "Experimental" line with intermediate reproductive performance levels. Whereas expression of the leptin receptor mRNA did not change in the theca, it clearly decreased with follicular differentiation in the granulosa of slow growing hens. In fast growing standard hens fed ad libitum and presenting significant reproductive dysfunction, the decrease was disrupted and dramatic up-regulation of granulosa cell expression of the leptin receptor was observed. On the other hand, feed restriction decreased the overall level of expression of the leptin receptor mRNA and restored the decrease with follicular growth. The level of expression of the leptin receptor probably modulates the action of leptin on follicular differentiation. Since blood leptin and other metabolic factors were not affected by the genotype or by nutritional state, the factors involved in the regulation of leptin receptor gene expression remain to be determined. This study demonstrates the involvement of leptin in the nutritional control of reproduction in birds. Leptin action on the ovary probably controls follicular hierarchy through the regulation of steroidogenesis.

## Background

The ovary of the mature hen contains a hierarchy of yellow yolky follicles and several thousand smaller follicles from which the large yolky follicles are recruited. The yellow follicles are arranged in a size hierarchy and are committed to ovulation. In each follicle, the granulosa cells are surrounded by theca tissue and are separated from it by a basement membrane. Each compartment of the largest yellow follicles (theca and granulosa cells) can be anatomically separated to follow the individual functions of these two ovarian compartments in follicle growth and differentiation. At an early stage of follicular development the small ovarian follicles produce estrogens and androgens. As follicles begin to sequester yolk their production of estrogens from theca cells decreases to become very low at ovulation. As follicles are recruited into the yolky follicular hierarchy, estrogen and androgen production by theca cells diminishes and production of progesterone by granulosa cells increases. The largest F1 yellow follicle then attains the highest progesterone production at the time of ovulation.

In most domestic animals, reproductive function is considerably affected by nutrition [1,2]. Various hormones, including growth hormone, insulin-like growth factors (IGFs) and insulin, have been proposed as potential mediators affecting reproductive function [3]. However, the interactions between the reproductive endocrine axis and the metabolic axis have not been clearly determined. Leptin represents also a good candidate for such reproductive-metabolic interactions. Leptin, the protein hormone synthesized and secreted mainly by adipose tissue, has primarily been shown to regulate food intake and energy expenditure (for review see [4-6]). Recent studies have demonstrated that leptin may also be involved in the regulation of reproductive mechanisms in human and rat ovaries [7-10]. Exogenous leptin can rescue reproductive function in ob/ob leptin-deficient mice that are not only obese but also infertile. This leptin action is independent from weight loss since feed restriction in ob/ob female mice fails to restore fertility [11]. Leptin can also advance the onset of puberty or at least reverse the delay caused by feed restriction in rodents [12,13]. In chickens, leptin attenuates the negative effects of fasting on ovarian function. Injections of leptin during fasting delays cessation of egg laying, attenuates regression of yellow hierarchical follicles, alters ovarian steroidogenesis and limits apoptosis [14]. Leptin exerts its effect by binding to a receptor which belongs to the cytokine receptor super-family [15]. The chicken leptin receptor has been cloned and sequenced [16,17]. Its expression at the level of the ovary [14,17] suggests that leptin might act directly on the ovary to regulate chicken reproductive function.

Standard broiler breeders have been submitted to high selection pressure on growth and feed efficiency. Male traits have been favoured resulting in poorer reproductive performances of the hens. As a result of such selection, broiler breeder hens are subject to metabolic disorders and reproductive dysfunction. Overfeeding during reproductive development is associated with the formation of excessive numbers of ovarian yellow follicles which can be arranged in multiple hierarchies, with increased production of unsettable eggs [18-21]. Severe feed restriction during rearing reduces the production of yellow follicles, the incidence of double ovulation and considerably improves the laying rate [19]. Up-regulation of yellow follicles has been related to excessive recruitment and rapid growth rate of follicles to maturity, especially under ad libitum feeding [19]. However, the mechanisms that regulate these processes are still not fully explained.

This study investigated the potential involvement of leptin and its receptor in ovarian abnormalities observed in broiler breeder hens fed ad libitum. We aimed to determine the evolution of leptin receptor gene expression in both granulosa and theca cells from the four largest yellow follicles of 32 week-old hens during the laying period. The effects of genotype and diet on plasma leptin levels and ovarian expression of the leptin receptor gene were also quantified. For this purpose standard broiler breeder hens fed ad libitum or feed-restricted were compared to a French "Label" genotype and a dwarf "Experimental" line. Compared to fast growing standard broiler breeders, the French "Label" is a dwarf, slow-growing broiler genotype and the "Experimental" line is a dwarf genotype with a growth potential of progeny chicks close to that of the standard broiler chicks. The "Experimental" line is specifically selected for reproductive traits and viability at partial expenses of growth performances. The "Label" line tolerates ad libitum feeding of breeders and does not have reproductive problems under ad libitum feeding whereas the «Experimental» line can be fed ad libitum on a low energy diet during the growing period but presents more reproductive problems than the Label genotype but less than the standard genotype fed ad libitum.

## Methods

### Animals

Three lines of broiler breeder hens supplied by Hubbard primary breeder (Chateaubourg, France) were used in this experiment. The S line is a standard fast growing broiler, the French Label (L) line is a slow growing broiler breeder strain used for the quality market and the experimental (E) line is a broiler breeder strain bearing the "dw" dwarf gene. This strain has a decreased need for rationing. S and L hens were given the same regime in accordance with Hubbard nutritional recommendations (2724 kcal/kg) in fine meal form. During the growing period (0–20 weeks), half of the S hens were feed restricted (SR) on the same diet in order to match a reference body weight curve provided par Hubbard primary breeder, the other half (SA) and all the L hens were fed ad libitum. The feed intake was equivalent to 37% of the SA group up to point of lay [22]. A special diet was designed for the E hens, consisting of a series of finely ground meal diets with a lower energy content (2550 kcal/kg). The interest of the E group is mainly practical, it represents an actual alternative for severely restricted standard broiler breeder hens [22]. Transition between the grower and breeder feed occurred at 20 weeks of age for the ad libitum-fed hens (L, SA and E). Transition occurred at the beginning of laying for the restricted hens that were then allowed ad libitum access to food.

At 32 weeks of age, blood samples were collected from 12 hens of each experimental group (SA, SR, E and L) and six hens were sacrificed by an overdose of pentobarbital (Sanofi-Santé Animale, Libourne, France). The ovaries and liver were immediately removed. Granula and theca compartments from the first (F1), second (F2), third (F3) and fourth (F4) largest ovarian yellow follicles were dissected as previously described [23]. Since SA birds presented a greater average number of yellow follicles per ovary (9.36, 8,00, 7.42, and 6.33 yellow follicles/ovary for the SA, SR, E and L hens respectively) and a higher proportion of pairs of yellow ovarian follicles undergoing simultaneous development [22], follicles were assigned to the same follicular rank when the difference of weight between two follicles was of 0.4 g or less. In that case, one follicle per pair was collected and dissected. Tissues were immediately snap frozen in liquid nitrogen and stored at -80°C until used for total RNA extraction. This experiment was carried out with due regard to the legislation governing ethical treatment of animals, and investigators were certificated by the French government to carry out animal experiments.

### RNA extraction and leptin receptor RT-PCR

Total RNA was extracted from liver, granulosa and theca cells using RNA InstaPure (Eurogentec, Angers, France) according to the manufacturer's recommendations. After DNAse treatment using Ambion's DNA-free kit (Clinisciences, Montrouge, France), 2 μg of total RNA were reverse-transcribed (RT) in a final volume of 20 μl using RNAse H^- ^MMLV reverse transcriptase (Superscript II, Invitrogen, Cergy Pontoise, France) and random hexamer primers (Promega, Charbonnières, France). cDNA was then diluted to 1:8.

For normal PCR amplification, five microliters of the RT reaction were amplified for 35 cycles in a 50 μl reaction volume containing 2.5 units of Taq DNA Polymerase (Amersham Biosciences, Orsay, France), 2.5 mM MgCl_2_, 0.2 mM dNTPs (Promega, Charbonnieres, France) and 0.2 μM of each forward and reverse primer. Leptin receptor forward (5'-GTC CAC GAG ATT CAT CCC AG-3') and reverse (5'-CCT GAG ATG CAG AGA TGC TC-3') primers were chosen according to the previously determined sequence of the chicken leptin receptor cDNA [17]. This pair of primers amplifies a 271 bp cDNA fragment located in the coding sequence of the extra-cellular domain. The amplification conditions were as follows: denaturation at 94°C for 30 sec, annealing at 60°C for 30 sec and primer extension at 72°C for 60 sec. After final extension at 72°C for 10 min, PCR products were resolved on 1.5% agarose gel containing ethidium bromide.

### Real-time RT-PCR

Real-time RT-PCR was performed as previously described [24]. Briefly, forward leptin receptor primer 5'-GCATCTCTGCATCTCAGGAAAGA-3' and reverse leptin receptor primer 5'-GCAGGCTACAAACTAACAAATCCA-3'(nucleotides 362 to 448 of the chicken leptin receptor cDNA sequence) [16,17] were designed to be intron-spanning to avoid co-amplification of genomic DNA using Primer Express Software (Applied Biosystems, Courtaboeuf, France). A 20 μl master mix containing 12.5 μl SYBR Green PCR Master Mix, 1 μl forward primer (300 nM), 1 μl reverse primer (300 nM) and 5.5 μl water was prepared to perform real-time PCR (Applied Biosystems, Courtaboeuf, France). Five microliters of cDNA dilution was added to the PCR Master Mix to a final volume of 25 μl. The following PCR protocol was used on the ABI Prism 7000 apparatus (Applied Biosystems, Courtaboeuf, France): initial denaturation (10 min at 95°C), followed by a two-step amplification program (15 sec at 95°C, followed by 1 min at 60°C) repeated 40 times. Quantification was performed using ABI integrated software as previously described [24]. 18S ribosomal RNA was chosen as the reference gene. The level of 18S RNA was determined using the Pre-developed TaqMan Ribosomal RNA control kit (Applied Biosystems, Courtaboeuf, France) according to the manufacturer's recommendations. The results were expressed as the leptin receptor mRNA/18S RNA ratio. Each PCR run included a no template control and replicates of control and unknown samples. Runs were performed in triplicate.

### Plasma lipid, glucose and hormone analyses

Total cholesterol, phospholipid, and triglyceride plasma concentrations were calculated with "Cholesterol RTU", "Phospholipides Enzymatique PAP 150", and "Triglycérides Enzymatique PAP 150" kits (bioMérieux, Charbonnières les Bains, France) according to the manufacturer's recommendations. Plasma glucose levels were measured by the glucose oxidase method using an automated analyzer. Plasma insulin levels were determined by a radioimmunoassay with a guinea pig anti-porcine insulin antibody using chicken insulin as the standard [25]. Plasma concentrations of leptin were determined by a multi-species leptin RIA kit (LINCO Research Inc, CliniSciences, Montrouge, France) according to the recommendations of the manufacturer.

### Statistical analysis

The results of plasma lipid, glucose and hormone analyses as well as leptin receptor mRNA expression in the liver were analyzed by one-way ANOVA and means were compared by Student Newman Keuls multiple comparison test. The effects of the groups of birds (E, L SA and SR), follicular rank and possible interaction on the logarithm of leptin receptor mRNA levels were tested by two-way ANOVA using the General Linear Model (GLM) procedure of SAS (SAS Institute, 1999. SAS User' Guide, Version 8 ed. SAS Institute Inc., Cary, NC). An additional effect of the subject was introduced into the model in order to take into account the fact that measurements of leptin receptor mRNA expression for the different follicular ranks were performed on the same animal. Pairwise comparisons of means for each significant effect of the ANOVA were performed by Scheffe test with the least means squares statement of the GLM procedure. The level of significance was set at *P < 0.05*.

## Results

### Leptin receptor mRNA expression in granulosa and theca cells

**Figure 1:**
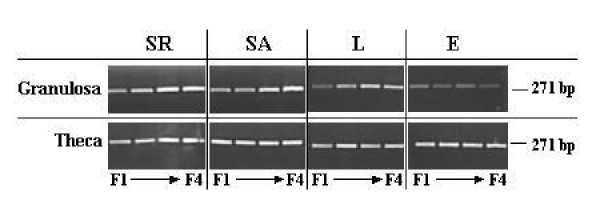
Amplification by RT-PCR of leptin receptor mRNA (271 bp) in granulosa and theca cells of the four largest yellow follicles (F1, F2, F3 and F4) inbroiler breeder hens from a dwarf Experimental line (E), a French Label line (L) and a Standard line fed ad libitum (SA) or restricted (SR).

We demonstrated the expression of leptin receptor mRNA in granulosa and theca cells from the three genotypes fed ad libitum or restricted for the S line. The expression of leptin receptor mRNA for both ovarian cells was detected in each hierarchical yellow follicle studied (F1 to F4) (Figure 1).

### Evolution of leptin receptor mRNA expression with follicular development

The evolution of expression of leptin receptor mRNA during follicle development was investigated in both granulosa and theca cells from F4 to F1 yellow follicles using real-time RT-PCR. Since leptin receptor mRNA levels did not follow a normal distribution (skewness of 5.48 and Kurtosis of 32.88), they were log transformed. The resulting distribution was closer to the normality with skewness of 0.87 and kurtosis of 0.69. Variance analysis was performed on transformed data.

**Figure 2:**
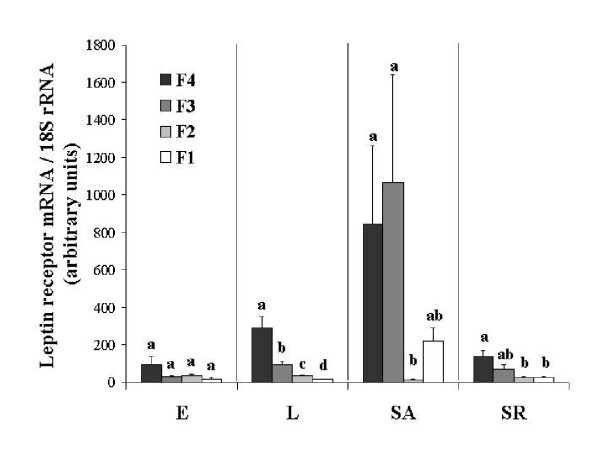
Relative levels of leptin receptor mRNA assessed by real time RT-PCR in the granulosa cells of the four largest yellow follicles (F1, F2, F3 and F4) in broiler breeder hens from a dwarf Experimental line (E), a French Label line (L) and a Standard line fed ad libitum (SA) or restricted (SR). The results were corrected by the corresponding levels of 18S rRNA. Data are expressed as mean ± SEM, n = 6. Bars with different letters are significantly different within groups (P < 0.05).

As shown in Table 1 and Figure 2, expression of leptin receptor mRNA in granulosa cells from the L genotype clearly decreased as the follicle developed. Significant statistical differences were measured between hierarchical yellow follicles at each stage. In the E line, expression of the leptin receptor mRNA decreased between F4 and F1 yellow follicles. However the high variability of the expression of leptin receptor mRNA in F4 follicles prevented the decrease from reaching statistical significance. Compared to the L line, the level of expression of the leptin receptor in the granulosa cells was lower in the E group, especially for the F4 and F3 yellow follicles but statistical significance was reached only for the F4 follicles (Table 1). In the S line fed ad libitum, expression of the leptin receptor in the granulosa was dramatically up-regulated. This up-regulation was clearly evident in F4 F3 and F1 follicles. Wide variability was also observed in F4 and F3 follicles. Feed restriction of the standard hens (SR) induced a general decrease in the expression of leptin receptor mRNA. The overall level of expression of the leptin receptor mRNA measured in the SR line was similar to that observed in the L and E lines. Compared to the SA birds, feed restriction has restored the decrease in the expression of leptin receptor mRNA with follicle development.

**Table 1:** Leptin receptor mRNA levels normalized to the level of 18S rRNA (expressed as arbitrary units) in the granulosa of ovarian F1, F2, F3 and F4 yellow follicles.

	**E**	**L**	**SA**	**SR**	**ANOVA**
F1	18.63 ± 5.26^a^	16.34 ± 2.26^a^	219.01 ± 72.23^b^	24.73 ± 4.20^a^	*P *< 0.0001
F2	34.60 ± 6.18^a^	32.78 ± 4.73^a^	11.57 ± 4.31^a^	26.89 ± 3.89^a^	NS
F3	29.78 ± 3.43^a^	92.80 ± 18.25^a^	1062.90 ± 580.82^b^	71.31 ± 20.92^a^	*P *< 0.001
F4	91 ± 48.85^a^	290 ± 57.93^b^	844 ± 420.56^b^	135.22 ± 32.26^a^	*P *< 0.01

Data are expressed as mean ± SEM (n = 6). Data with different letters are significantly different between groups.

**Figure 3:**
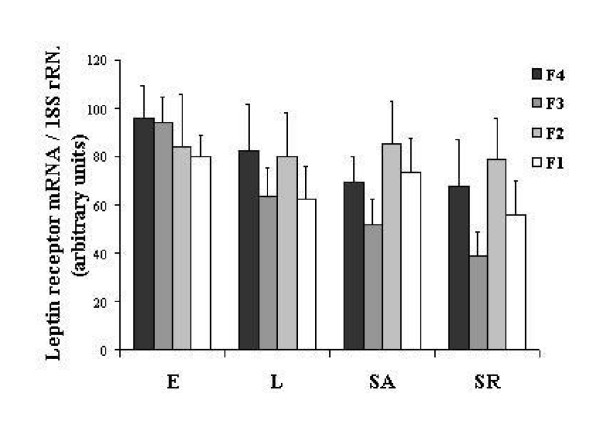
Relative levels of leptin receptor mRNA assessed by real time RT-PCR in the theca cells of the four largest yellow follicles (F1, F2, F3 and F4) in broiler breeder hens from a dwarf Experimental line (E), a French Label line (L) and a Standard line fed ad libitum (SA) or restricted (SR). The results were corrected by the corresponding levels of 18S rRNA. Data are expressed as mean ± SEM, n = 6.

Expression of leptin receptor mRNA in the theca cells remained stable during yellow follicle development, whatever the group of birds considered. Statistical analysis did not reveal any difference in leptin receptor mRNA expression between the 4 groups of birds (Figure 3).

### Expression of leptin receptor mRNA in the liver

**Figure 4:**
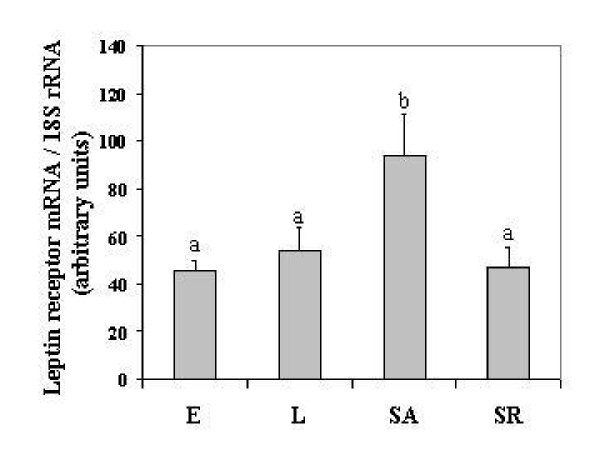
Relative levels of leptin receptor mRNA assessed by real time RT-PCR in the livers of broiler breeder hens from a dwarf Experimental line (E), a French Label line (L) and a Standard line fed ad libitum (SA) or restricted (SR). The results were corrected by the corresponding levels of 18S rRNA. Data are expressed as mean ± SEM, n = 6. Bars with different letters are significantly different (P < 0.05).

In the liver, the expression of leptin receptor mRNA was up-regulated in S birds fed ad libitum. Feed restriction of S birds restored the level of expression of leptin receptor mRNA similar to that measured in the E and L birds (Figure 4).

### Plasma lipid and glucose and hormone concentrations

At 32 weeks of age plasma leptin and insulin concentrations were found to be similar in the three genotypes. Food restriction of the standard hens did not alter plasma leptin, glucose or insulin levels (Table 2). Triglyceride, cholesterol and phospholipid levels were also measured. Cholesterol and phospholipid levels were not affected by genotype or diet. On the other hand, triglyceride levels seemed to be affected by the genotype. Lower triglycerides levels were measured in Standard birds (SA, SR). However, statistical significance was reached only for the restricted SR birds.

**Table 2:** Lipid and hormone plasma levels.

	**E**	**L**	**SA**	**SR**
Leptin (ng/ml)	1.68 ± 0.17	2.08 ± 0.23	2.06 ± 0.19	1.97 ± 0.21
Insulin (pmol/ml)	118.6 ± 17.9	101.1 ± 12.0	129.7 ± 29.7	128.3 ± 16.3
Glucose (mmol/l)	10.99 ± 0.39	10.60 ± 0.22	11.10 ± 0.22	10.71 ± 0.33
Triglycerides (mmol/l)	31.36 ± 1.89	33.48 ± 1.63	25.01 ± 3.41	19.15 ± 3.34*
Phospholipids (mmol/l)	12.10 ± 0.29	12.75 ± 0.47	10.53 ± 1.14	11.04 ± 1.24
Cholesterol (mmol/l)	9.16 ± 0.39	9.48 ± 0.40	8.40 ± 0.63	9.07 ± 0.77

Data are expressed as mean ± SEM (n = 12). * indicates statistical difference (*P *< 0.05)

## Discusssion

Several studies conducted on theca and granulosa cells have shown that leptin may have direct negative effects on ovarian steroidogenesis in various mammalian species. Leptin inhibits insulin-induced progesterone and 17β-estradiol production by isolated bovine granulosa cells [26] and impairs the hormonally-stimulated in vitro release of 17β-estradiol by rat granulosa cells [27]. In granulosa cells from fertile women, leptin inhibits FSH and IGF-I stimulated estradiol production [28,29] Since leptin has a more potent inhibitory action of insulin-induced aromatase activity of granulosa cells from small than large follicles, it has been proposed that the numbers of leptin receptors in granulosa cells might decrease as follicles develop in order to make mature Graafian follicles less sensitive to the negative action of leptin [26,30]. As shown in this study and in previous reports [14,17], the leptin receptor was expressed in the hen ovary in both granulosa and theca cells, suggesting a direct action of leptin at the level of the ovary. It seemed that leptin might affect ovarian steroidogenesis in laying hens during fasting [14] but the involvement of leptin on steroidogenesis during normal follicle development remained to be determined. In this study we demonstrated that the direct action of leptin on the ovary might be modified during follicle development since the level of expression of its receptor clearly decreased during maturation of yellow follicles. This decrease was particularly evident in slow growing broiler breeder hens from the "Label" genotype and from the feed-restricted standard line. Given that fast growing chickens (ad libitum-fed standard and Experimental broiler breeder hens) have the highest reproductive problems, genetic or nutritional control of the growth rate might regulate ovarian leptin receptor gene expression and improve reproductive function. Such evolution of receptor expression in the follicular hierarchy has previously been shown for the FSH receptor. FSH-stimulated steroidogenesis declined during follicle maturation and was associated with a decrease in FSH receptor numbers [31]. Conversely, the expression of mRNA encoding the IGF-I receptor and the related efficacy of binding of IGF-I to granulosa cells increased as the follicle matured [32,33]. Since leptin receptor gene expression was modified during follicle development, leptin might also be involved in regulation of the follicular hierarchy and onset of preovulatory steroidogenesis, as has been proposed for gonadotrophins and growth factors including FSH and IGF-I.

Unlike mammals, progesterone in chickens is synthesized and secreted mainly by granulosa cells whereas theca cells generate estradiol [34]. Progesterone produced by granulosa cells from mature follicles provides the positive feedback necessary to stimulate a preovulatory surge of LH [35]. IGF-I has been involved in the regulation of ovarian steroidogenesis in both mammals and birds. IGF-I stimulates progesterone production from avian granulosa cells [36] whereas it up-regulates estradiol from mammalian granulosa cells [27-29]. Since leptin is considered to be an inhibitor of insulin and IGF-I action on steroidogenesis in mammals, leptin might have similar negative action in birds. Thus, the decrease in its receptor in the granulosa suggests that the inhibiting action of leptin would decrease during follicle development and consequently favours the stimulatory effect of gonadotrophins and IGF-I on follicular maturation. This hypothesis is also consistent with the weaker steroidogenic response of granulosa cell culture of ad-libitum fed standard broiler breeder hens when stimulated by IGF-I compared to granulosa cell culture from feed-restricted birds [37]. Moreover, Onagbesan et al (2004) demonstrated in an experiment similar to that performed in the present study and using the same genotypes that plasma progesterone levels were clearly affected in SA birds. They demonstrated that plasma progesterone levels remained relatively stable between 25 and 37 weeks of age in the E, L and SA birds with a significant lower level in the SA birds (2.2 ± 0.62 ng/ml for SA birds compared to 3.9 ± 0.36 and 4.2 ± 0.54 for L and E birds respectively). In restricted standard birds, plasma progesterone levels dramatically increased and reached values (3.8 ± 0.26 ng/ml) closed to that measured in the E and L lines (Onagbesan et al., 2004, data from progesterone levels were personal communication from Dr Onagbesan, Catholic University of Leuven, Belgium).

The erratic pattern of oviposition in standard broiler breeder hens fed ad libitum has been previously demonstrated to be related to abnormal maturation of steroidogenesis, particularly in the two largest yellow follicles [35]. Since F2 and F1 yellow follicles presented similar endocrine profiles, the preovulatory surge of LH probably triggers ovulation of the two largest follicles [21,36]. In this study we have shown that ad libitum feeding of broiler breeder hens dramatically up-regulated expression of the leptin receptor in the granulosa cells of yellow follicles and changed the evolution of expression of this receptor with follicle development. These results suggest a strong action of leptin on the ovaries of ad libitum fed birds. Feed restriction reduced the level of expression of the leptin receptor and on the whole restored the evolution of expression of the receptor with follicle maturation. Since ad libitum feeding affects the hierarchical endocrine order of the follicles, as a potential inhibitor of hormonally induced avian steroidogenesis leptin represented a good candidate to explain the affects of follicular hierarchy. Up-regulation of the expression of the leptin receptor gene was also demonstrated in the liver. This up-regulation may be related to the control of lipogenesis. The liver plays a key role in lipid metabolism and lipogenesis in avian species [38,39] and the standard broiler breeder hens were the fattest birds of this experiment.

Since expression of its receptor was dramatically up-regulated in SA hens, leptin probably played an important role in the increased number of large yellow follicles and abnormal follicle hierarchy. However the factors involved in regulation of the expression of the leptin receptor within the hen ovary remains to be determined. Among the plasma hormones and lipids analyzed in this study only triglycerides were found to be different between strains, with a lower level in the restricted standard broiler breeder hens that were also the leanest birds. Down regulation of the expression of the leptin receptor by homologous and heterologous signals have previously been demonstrated in both mammals [40,41] and chickens [24]. Leptin and insulin are able to down-regulate expression of the chicken leptin receptor in vitro. In the present study, plasma leptin and insulin levels were similar for each genotype and were not altered by feed restriction in the standard genotype. We have previously demonstrated that during the first 5 weeks of age, plasma leptin levels remained relatively stable in both broiler and layer chicken despite increased body weight [42]. However the absence of leptin levels differences may be related to the fact plasma leptin levels were measured at 32 weeks of age. SR birds were relaxed at the start of lay, switched to breeding feeding and allowed ad libitum access as the other groups of birds. We therefore suggested that leptin and insulin are probably not involved in the regulation of ovarian leptin receptor gene expression in ad libitum or feed-restricted standard broiler breeder hens.

Evidence of the regulation of expression of the leptin receptor gene in the granulosa related to follicle maturation and nutritional state strongly suggest that leptin played an important local and sequential role in the dysfunction of the follicular hierarchy observed in standard broiler breeder hens fed ad libitum. This study suggests that the level of expression of the leptin receptor regulates the action of its ligand at the level of the ovary. This provides an interesting perspective to understanding the physiological role of leptin in the ovary.
